# Utilization of Carrier-Frequency Offset Measurements in UWB TDoA Positioning with Receiving Tag

**DOI:** 10.3390/s23052595

**Published:** 2023-02-26

**Authors:** Josef Krška, Václav Navrátil

**Affiliations:** Department of Radio Engineering, Faculty of Electrical Engineering, Czech Technical University in Prague, 166 27 Praha, Czech Republic

**Keywords:** ultra-wideband (UWB), time difference of arrival (TDoA), indoor localization, synchronization, carrier frequency offset (CFO), Levenberg–Marquardt algorithm, clock drift compensation

## Abstract

High-capacity impulse-radio ultra-wideband (IR-UWB) indoor localization systems are typically based on the time difference of arrival (TDoA) principle. When the fixed and synchronized localization infrastructure, the anchors, transmit precisely timestamped messages, a virtually unlimited number of user receivers (tags) are able to estimate their position based on differences in the time of arrival of those messages. However, the drift of the tag clock causes systematic errors at a sufficiently high magnitude to effectively deny the positioning, if left uncorrected. Previously, the extended Kalman filter (EKF) has been used to track and compensate for the clock drift. In this article, the utilization of a carrier frequency offset (CFO) measurement for suppressing the clock-drift related error in anchor-to-tag positioning is presented and compared to the filtered solution. The CFO is readily available in the coherent UWB transceivers, such as Decawave DW1000. It is inherently related to the clock drift, since both carrier and timestamping frequencies are derived from the identical reference oscillator. The experimental evaluation shows that the CFO-aided solution performs worse than the EKF-based solution in terms of accuracy. Nonetheless, with CFO-aiding it is possible to obtain a solution based on measurements from a single epoch, which is favorable especially for power-constrained applications.

## 1. Introduction

UWB impulse radio technology (IR-UWB) compliant with the IEEE 802.15.4 standards is capable of providing accurate localization in short-range scenarios. Such systems are suitable for indoor locations or other areas where other localization systems such as GNSS (Global Navigation Satellite Systems) are unreliable or unavailable [1,2]. Generally, the user devices (tags) are localized using measurements between them and fixed infrastructure devices with known positions (anchors). The approaches to UWB real-time localization may be based on one of two measurement methods: two-way ranging (TWR) or time difference of arrival (TDoA) [3].

The former, TWR-based localization, exploits range measurements from tag to several anchors, and estimates the tag position using multilateration. The TWR, however, requires bi-directional communication between the devices, and thus is relatively slow. Typically, double-sided TWR (DS-TWR) with three-message exchange is used [4] in order to mitigate the clock drift effect. Alternatively, single-sided TWR (SS-TWR) may be used provided that the drift-related error is mitigated by other means [5]. Obtaining a set of TWR measurements between tag and several anchors is rather time-consuming. Even though some reduction in the necessary time is achievable with optimization of the message scheduling [6] and relaxation of the constraints on reply delay symmetry by AltDS-TWR [7], no more than lower tens of position fixes per second are achievable [8].

The TDoA localization utilizes uni-directional communication between anchors and tag and thus is able to provide more frequent position fixes (up to several thousands fixes per second [9]) than the TWR-based approach. Nonetheless, the anchors have to share a common timescale or employ alternative ways of mutual bias mitigation that their free-running clocks generally have. The synchronization of the UWB anchors by means of Kalman filtering outperforms the other methods of UWB transceiver synchronization [10]. In [11], it is shown that it is beneficial to track clock drift rate in addition to clock bias and drift, and that sub-nanosecond accuracy of synchronization is achievable even during device warm-up. The measurement analysis in [12] proves that accuracy of the method is achieved even when the timing information is relayed via several anchors. The methods listed in [13,14] do not use Kalman filter explicitly; however, bias and drift estimates are obtained and utilized. In [15], a combination of a linear interpolation algorithm and Kalman filter is used. Typically, one of the anchors is used as the source of the master clock [10,11,13,14]. However, a less practical approach relying on a reference tag is also possible [16].

The synchronization-free methods, such as Sequential TDoA (S-TDoA) [17] rely on quick retranslation of the messages by the anchors; consequently, the positioning accuracy is severely limited by the retranslation delay and the clock performance. The research reported in [18] is claimed to be synchronization-free. However, the authors use Kalman filtering for estimation of the clock drift.

The TDoA localization can be performed in two directions. In the first case, the tag transmits a single blink message that is captured by the synchronized anchors. The position of the tag is then computed from the reception time differences at the infrastructure side. We will further denote this approach as the T2A (tag to anchor), which is equivalent to term Monitor-based localization (MBL) used in the survey [19]. Alternatively, the tag can receive precisely timed messages sent by the anchors and compute its own position from the time differences of arrival. This approach will be denoted as A2T (anchor to tag), and is consistent with device-based localization (DBL) used in [19].

The T2A approach is the simpler one to utilize, since the relative clock drift between the tag and infrastructure is not causing any problems and since the tag sends only one message, which is captured by all the anchors in range. However, the number of localized tags is limited by the number of messages that the anchors are able to receive.

When adopting the A2T approach, the messages from multiple anchors have to be received at the tag. This approach is similar to GNSS, where the ranging signals transmitted by the satellites are received by the user; as a consequence, unlimited number of tags can receive the signals and compute their own position. However, unlike the GNSS signals, the IR-UWB signals are not continuous and do not utilize code-division multiplexing; therefore, it is not possible to receive and separate them in a similar fashion.

If the UWB messages are transmitted at the (almost) same time, the tag cannot receive them in a regular way, the time of arrival differences have to be deduced from the channel impulse response (CIR) estimated by the receiver. Such an approach is adopted e.g., in [20,21,22]. Problems with identification of the individual transmitters of the signals observed in CIR and masking of pulses from similarly distant anchors are inherent to this approach. Moreover, the data transfer is completely neglected as well.

Without the utilization of the CIR, it is necessary to temporally separate the messages from the individual anchors, since only one message can be received by the tag at any instant. Practically, a millisecond-level separation of the transmitting times is necessary to mitigate the collisions. The delay due to separation is then compensated in the tag. Nonetheless, with up to ±20 ppm drift of the UWB transceivers [23], the relative time interval error between two devices can reach up to 40 ns per millisecond of message separation. When left uncorrected, such measurement errors result in errors of several tens of meters and render positioning barely possible. The S-TDoA approach [17] reduces the error by using more stable and accurate clocks in the UWB devices and minimizing the separation delays. The S-TDOA approach is limited to utilizing differences between adjacent anchors in the transmission sequence, which exhibit minimal drift-related errors. In research by [24,25], it has been shown that it is possible to accurately estimate the relative clock drift between the tag and the synchronized anchors by means of Kalman filtering. The knowledge of the drift is used to compensate for the time interval error between the tag and anchor. Moreover, the method [25] allows simultaneous operation of the T2A and A2T modes. Nonetheless, at least two measurement epochs are necessary to estimate the clock drift, which is undesirable for devices with limited energy available.

However, coherent UWB transceivers, such as DW1000 [26], are able to estimate carrier frequency offset (CFO). Since the carrier frequency is derived from the same 38.4 MHz base frequency reference as the 63.8976 GHz sampling and timestamping clock, the relative clock drift between receiver and transmitter is available as properly scaled CFO. The CFO has already been successfully used for compensation of drift-related errors in TWR-based positioning, and in T2A asynchronous-TDOA [5]. According to the authors’ knowledge, the CFO has not been previously used in A2T TDoA positioning. The research presented in this article proves that A2T TDoA positioning can be performed based on a single set of messages from the synchronized anchors when using CFO estimates.

Firstly, we will briefly recapitulate the anchor synchronization algorithm. Secondly, two ways of obtaining the CFO will be presented and briefly compared. In the following section, the CFO-aided A2T TDOA algorithm will be described. Section 5 is devoted to the experimental evaluation of the presented method with respect to ground truth and the performance comparison with the Kalman-filter-based A2T TDoA method from [25]. The article concludes with a discussion of the results and summary.

## 2. Wireless Anchor Network Synchronization

Each of the UWB transceivers (either tag or anchor) has its own free-running reference clock. Rather than steering the clock physically, it is more feasible to track and predict the clock bias Δt[k], drift $\Delta \dot{t}\left[k\right]$ and drift rate $\Delta \ddot{t}\left[k\right]$ with respect to the chosen master anchor clock [11]. A linear Kalman filter is utilized; the state vector x[k], where *k* is the index of epoch, is defined as

(1) $$x\left[k\right]={\left[\begin{array}{ccc} \Delta t[k] & \Delta \dot{t}\left[k\right] & \Delta \ddot{t}\left[k\right] \end{array}\right]}^{T}.$$

The discrete process model of the synchronization Kalman filter is described by matrix F.The time-update of the state vector x and its covariance matrix P can be written as:

(2) $$\begin{array}{cc} x^{-}\left[k\right] & =F\left[k\right]x^{+}\left[k-1\right] \end{array}$$

(3) $$\begin{array}{cc} P^{-}\left[k\right] & =F\left[k\right]P^{+}\left[k-1\right]F{\left[k\right]}^{T}+Q\left[k\right] \end{array}$$

(4) $$\begin{array}{cc} F[k] & =\left[\begin{array}{ccc} 1 & T[k-1] & \frac{1}{2}T{\left[k-1\right]}^{2}\\ 0 & 1 & T[k-1]\\ 0 & 0 & 1 \end{array}\right] \end{array}$$

(5) $$\begin{array}{cc} Q[k] & =\left[\begin{array}{ccc} T\left[k-1\right]\;\sigma_{x0}^{2} & 0 & 0\\ 0 & T\left[k-1\right]\;\sigma_{x1}^{2} & 0\\ 0 & 0 & T\left[k-1\right]\;\sigma_{x2}^{2} \end{array}\right]. \end{array}$$

Symbol T[k−1] is the time passed between epochs *k* and k−1; Q denotes the process noise matrix, whose diagonal elements should be tuned to match the performance of the particular clocks. It should be noted that the + and − superscripts indicate a posteriori and a priori values in the Kalman filter.

The bias (time offset) between master and slave clock $\Delta t^{\left[\mathrm{MS}\right]}\left[k\right]$ is observed by means of differentiating the transmission and reception timestamps and correcting for the master-to-slave propagation delay and equipment delays in the transmitter and the receiver. The bias is used as the measurement of the Kalman filter in the particular epoch y[k]. Therefore,

(6) $$y\left[k\right]=\Delta t^{\left[\mathrm{MS}\right]}\left[k\right]=t_{\mathrm{Rx}}^{\left[S\right]}\left[k\right]-t_{\mathrm{Tx}}^{\left[M\right]}\left[k\right]-\tau_{\mathrm{Tx}}^{\left[M\right]}-\tau_{\mathrm{Rx}}^{\left[S\right]}-\frac{r_{\mathrm{MS}}}{c_{0}},$$

where $t_{\mathrm{Tx}}^{\left[M\right]}\left[k\right]$ is the transmission timestamp in the master timescale (denoted by superscript [M]), $t_{\mathrm{Rx}}^{\left[S\right]}\left[k\right]$ is the reception timestamp in slave timescale, $\tau_{\mathrm{Tx}}^{\left[M\right]}$ denotes transmission equipment and antenna delay in master transceiver and $\tau_{\mathrm{Rx}}^{\left[S\right]}$ denotes reception equipment and antenna delay in slave transceiver. Symbol $r_{\mathrm{MS}}$ stands for the geometric distance of the master and slave antenna phase centers and $c_{0}$ is the propagation velocity, i.e., speed of light. Note that line of sight (LOS) between master and slave anchor is presumed.

The covariance matrix r of the measurement is indeed a scalar value. According to [10] the timestamp noise is Gaussian with 150 ps standard deviation. Two timestamps are combined in the bias measurement; from our practical experience it is reasonable to assume r=(250 ps${)}^{2}$.

Since the clock bias is also the first element of the state vector, the measurement model matrix is trivial, indeed; $H=\left[\begin{array}{ccc} 1 & 0 & 0 \end{array}\right]$. The filter measurement update follows equations

(7) $$\begin{array}{cc} z[k] & =y\left[k\right]-Hx^{-}\left[k\right] \end{array}$$

(8) $$\begin{array}{cc} S[k] & =HP^{-}\left[k\right]H^{T}+r\left[k\right] \end{array}$$

(9) $$\begin{array}{cc} K[k] & =P^{-}\left[k\right]H^{T}{\left(S\left[k\right]\right)}^{-1}. \end{array}$$

(10) $$\begin{array}{cc} x^{+}\left[k\right] & =x^{-}\left[k\right]+K\left[k\right]z\left[k\right]\\ P^{+}\left[k\right] & =\left(I-K[k]H\right)P^{-}\left[k\right]{\left(I-K[k]H\right)}^{T} \end{array}$$

(11) $$\begin{array}{cc} \; & +K\left[k\right]r\left[k\right]K^{T}\left[k\right], \end{array}$$

where z denotes innovation, S is innovation covariance matrix, K is the Kalman gain and I is the identity matrix of appropriate size. It is worth noting that r, and S are scalars, and thus the matrix inverse in (9) becomes a scalar division.

For the positioning purpose it is necessary to convert the local slave time to the master time. Prediction based on the Kalman filter state vector is performed in order to obtain the bias $\Delta t^{\left[\mathrm{MS}\right]}\left(t_{b}\right)$ between master and slave time at any instant shortly after the last measurement update; it is also possible to obtain the variance:

(12) $$\begin{array}{cc} \Delta t^{\left[\mathrm{MS}\right]}\left(t_{b}\right) & \approx F_{b}\left(t_{b}\right)x^{+}\left[k\right] \end{array}$$

(13) $$\begin{array}{cc} \mathrm{var}\left(\Delta t^{\left[\mathrm{MS}\right]}\left(t_{b}\right)\right) & \approx F_{b}\left(t_{b}\right)P^{+}\left[k\right]F_{b}{\left(t_{b}\right)}^{T}+\left(t_{b}-t\left[k\right]\right)\sigma_{x0}^{2}. \end{array}$$

Symbol $t_{b}$ denotes the time of prediction (expressed in local, slave time). The prediction matrix $F_{b}$ is based on the first line of the process model matrix

(14) $$F_{b}\left(t_{b}\right)=\left[\begin{array}{ccc} 1 & t_{b}-t\left[k\right] & \frac{1}{2}{\left(t_{b}-t\left[k\right]\right)}^{2} \end{array}\right],$$

where t[k] is the nearest (latest) synchronization epoch. The timestamp in the master time and its variance is available as

(15) $$\begin{array}{cc} t_{b}^{\left[M\right]} & \approx t_{b}^{\left[S\right]}-\Delta t^{\left[\mathrm{MS}\right]}\left(t_{b}\right) \end{array}$$

(16) $$\begin{array}{cc} \mathrm{var}\left(t_{b}^{\left[M\right]}\right) & \approx \mathrm{var}\left(t_{b}^{\left[S\right]}\right)+\mathrm{var}\left(\Delta t^{\left[\mathrm{MS}\right]}\left(t_{b}\right)\right). \end{array}$$

It is obvious that the accuracy of such correction is deteriorating with $\left(t_{b}-t\left[k\right]\right)$ term, i.e., the time passed since the last synchronization message reception. Naturally, the variance reflects the deterioration.

The repetition interval between synchronization messages (i.e., measurement updates) is a trade-off between accuracy and airtime utilization. In [11], it is shown that error bellow 500 ps (corresponds to 15 cm in range) is achievable in 95% of epochs, when 400 ms message interval is used. It is worth noting that under assumption of normal distribution the standard deviation is approximately half of the value, i.e., 250 ps. Thorough description and experimental evaluation of this algorithm and the way of chaining multiple synchronization segments is available in [11,12].

## 3. Carrier Frequency Offset Measurement

Both the timestamping clock and the carrier frequency are derived from a single reference. Consequently, the timestamping clock drift is equal to the relative carrier frequency offset (CFO). Two ways of obtaining absolute value of the CFO from DW1000 chips are presented in [26], however, the relative CFO is of interest. Therefore, the equations provided here are slightly modified.

### 3.1. Receiver Time Tracking Offset

The first option of obtaining CFO is utilizing the Receiver Time Tracking Offset (RTTO) values, reported by the receiver for each message received, and Receiver Time Tracking Interval (RTTI). The relative offset $\nu_{\mathrm{RTTO}}$ is obtained as [26]

(17) $$\nu_{\mathrm{RTTO}}=\frac{\mathrm{RTTO}}{\mathrm{RTTI}}.$$

Note that the dimensionless carrier offset fraction given from (17) can be scaled by $10^{6}$ to obtain the value in ppm (parts per million). The RTTI is dependent on the pulse-repetition frequency (PRF) used; for 64 MHz PRF it holds RTTI = 0x1FC0000 [26]. Therefore, the resolution of the RTTO-based CFO estimate is 0.03 ppm. The RTTO-based CFO is not accurate, however, the value is included in the double-buffer swinging set of the receiver, and thus it is not necessary to pause receiving of the messages.

### 3.2. Carrier Integrator

The second option of estimating CFO is based on carrier integrator value, which is also reported for each message [26]. Unlike RTTO, the carrier integrator value $C_{\mathrm{int}}$ is not featured within double-buffer swinging set, hence the receiver has to be stopped until $C_{\mathrm{int}}$ value is read. On the other hand, the carrier-integrator-based CFO estimate $\nu_{\mathrm{CINT}}$ is substantially more accurate than the RTTO-based value. The relative drift estimate is computed as [26]

(18) $$\nu_{\mathrm{CINT}}=-\frac{C_{\mathrm{int}}\;\cdot \;2^{-17}}{2\;\cdot \;N_{s}}\frac{f_{s}}{f_{c}},$$

where number of samples is either $N_{s}=1024$ for 850 kbit s${}^{-1}$ bitrate and 6.8 Mbit s${}^{-1}$, or $N_{s}=8192$ for 110 kbit s${}^{-1}$. In our case the carrier frequency is $f_{c}=3.9936\;\mathrm{GHz}$, the sampling frequency is $f_{s}=998.4\;\mathrm{MHz}$. Since the 6.8 Mbit s${}^{-1}$ bitrate is used in our devices, the resolution of such measurement is 0.93 ppb.

### 3.3. CFO Measurement Accuracy

Thorough evaluation of the stochastic parameters of the CFO estimates is beyond the scope of this article, however, we provide a brief analysis based on a 57 min long concurrent measurement of $\nu_{\mathrm{RTTO}}$ and $\nu_{\mathrm{CINT}}$. The measurement was performed between two stationary transceivers, messages were transmitted with 100 ms period. The drift estimate from the Kalman-filter-based synchronization algorithm (described in previous section) was used as the reference. During the measurement, the devices were warming up from approximately 15 ${}^{\circ }$C to 46 ${}^{\circ }$C on-chip temperature; consequently, the clock drift was changing during the first 35 min of the test, then it became almost constant.

**Figure 1:**
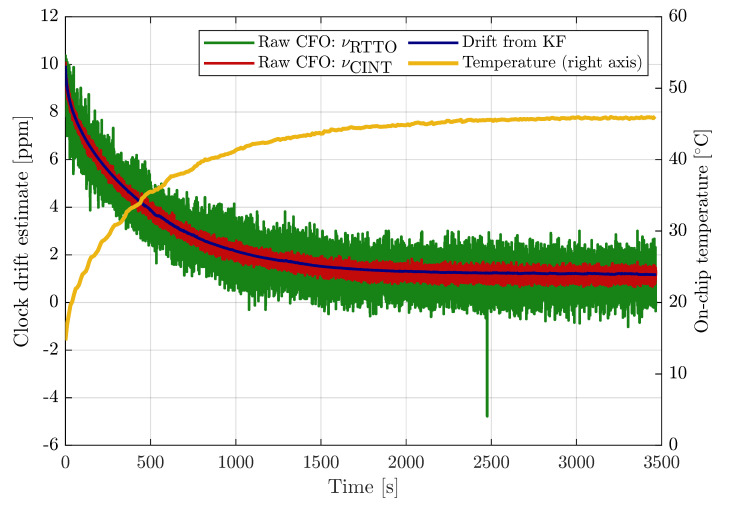
Raw measurements of relative CFO based on RTTO (green) and $C_{\mathrm{int}}$ (red), drift estimated by Kalman filter (blue) included for comparison. Moving average of receiver temperature (yellow) corresponds to the right axis.

The raw relative CFO estimates based on RTTO and carrier integrator are provided in Figure 1. Obviously, the RTTO-based CFO ($\nu_{\mathrm{RTTO}}$) is significantly noisier than the $C_{\mathrm{int}}$-based CFO ($\nu_{\mathrm{CINT}}$); the accuracy of both CFO estimates is clearly not limited by the resolution. The clock drift estimated by the Kalman filter ($\Delta \dot{t}\left[k\right]$, see (1)) is smooth in comparison with the CFO estimates. On the right axis of Figure 1 is the on-chip temperature of the device; moving average with 101-sample symmetric window has been applied in order to improve readability. The dependency of clock drift and temperature is apparent.

**Figure 2:**
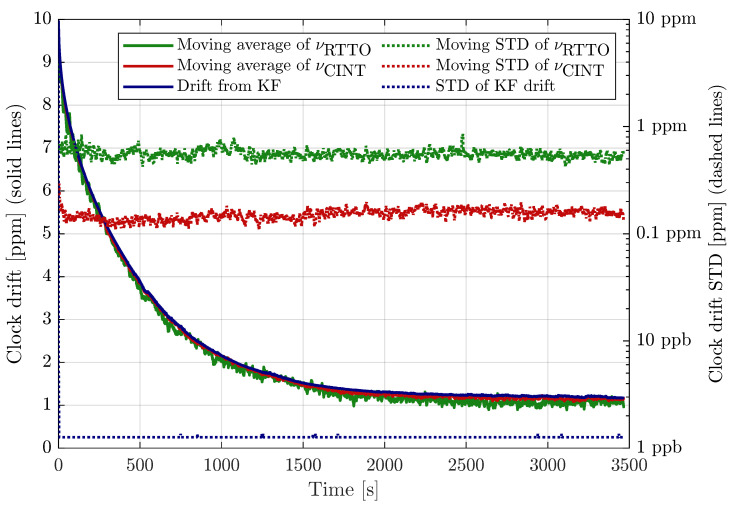
Moving average (solid lines, left axis) and moving STD (dashed lines, right axis) of relative CFO based on RTTO and $C_{\mathrm{int}}$, drift estimated by Kalman filter and its variance included for comparison.

Figure 2 presents moving averages and moving standard deviations (STD) of the CFO estimates. For both metrics 101-sample symmetric window has been applied in order to avoid lag. In other words, the averaged value is based on interval spanning 5 s before and 5 s after the current measurement. Both CFO moving averages are almost always visibly lower than the drift estimated by the EKF, which suggests that the estimates are slightly biased. We acknowledge that CFO-estimate bias has been observed also on longer measurements under constant-temperature conditions.

The moving standard deviations in Figure 2 are compared to the standard deviation of the Kalman filter drift estimate, which is deduced from the KF-state a posteriori covariance matrix ($P^{+}\left[k\right]$). The KF-based estimate STD is below 1.5 ppb once it converges within a few measurements. The moving STD of RTTO-based CFO estimate is between 0.4 ppm and 0.8 ppm, the $C_{\mathrm{int}}$-based CFO is approximately 4-times more accurate; its moving STD is mostly between 0.1 ppm and 0.2 ppm.

It is also worth mentioning that results in [5] suggest approximately 8-times better accuracy of CFO than experienced for $C_{\mathrm{int}}$-based CFO during our tests. As no quantification of accuracy and limited information about configuration is provided in [5], it is assumed that 110 kbit s${}^{-1}$ bitrate was used, and therefore the 8-times higher $N_{s}$ value in the denominator of (18) is the reason for such difference in accuracy.

Since the CFO estimates are approximately two orders of magnitude less accurate than the Kalman filter drift estimate, there is no advantage in utilizing the CFO measurement as an additional measurement input to the synchronization filter regardless the source of the CFO.

### 3.4. CFO of Synchronized Anchors

The local (physical) clock of a transceiver is synchronous with the master time only for the master anchor. In all other transceivers (synchronization slaves or relays) the timestamps from free-running local clock are corrected to the master time as described in Section 2. The relative CFO (ν) is measured between the local clocks of the transmitter and receiver, thus, in order to obtain the drift estimate (i.e., relative CFO) with respect to the master clock, it is also necessary to correct for the drift of the transmitter with respect to the master clock.

The normalized frequency of the slave local clock can be described as $\left(1+\Delta {\dot{t}}^{\left[MS\right]}\right)$, where $\Delta {\dot{t}}^{\left[MS\right]}$ is the clock drift between master and slave anchor (the accurate estimate from the KF). The tag is able to obtain the relative CFO between local clock of the slave anchor and its own local clock, which is denoted by $\nu^{\left[ST\right]}$. For the normalized frequency of the tag local clock with respect to the master clock $\left(1+\nu_{T}^{\left[M\right]}\right)$ it holds

(19) $$\left(1+\nu^{\left[MT\right]}\right)=\left(1+\nu^{\left[ST\right]}\right)\;\cdot \;\left(1+\Delta {\dot{t}}^{\left[MS\right]}\right)\approx 1+\nu^{\left[ST\right]}+\Delta {\dot{t}}^{\left[MS\right]}.$$

Such approximation is valid when both $\nu^{\left[ST\right]}\ll 1$ and $\Delta {\dot{t}}^{\left[MS\right]}\ll 1$. Due to requirements on clock accuracy in [23] magnitude of both terms should be below 4 × 10^−5^; therefore, the $\nu^{\left[ST\right]}\times \Delta {\dot{t}}^{\left[MS\right]}$ term omitted in the approximation should not be higher than 1.6 × 10^−9^, i.e., negligible in comparison with the CFO estimate accuracy. As a consequence, the clock drift (and CFO) estimates may be treated as additive. By a trivial modification of (19) it is possible to obtain a simple formula for CFO estimate between tag and master anchor:

(20) $$\nu^{\left[MT\right]}\approx \nu^{\left[ST\right]}+\Delta {\dot{t}}^{\left[MS\right]}.$$

## 4. CFO-Aided TDoA Localization

The A2T TDoA positioning, as described in [25], estimates the position of a tag from the received messages sent by the surrounding anchors. It is worth mentioning that the structure and content of the positioning messages is identical to the anchor synchronization messages. As a consequence, the tag is able to parasite on the anchor synchronization messages; it is sufficient that each anchor transmits a single message per epoch.

The UWB devices are able to receive only a single message at the time. Consequently, it is necessary to avoid message collisions when performing TDoA positioning with multiple transmitting anchors and receiving tags. Within a single frequency channel it is possible to separate the messages by transmitting them in non-overlapping time slots. The measured time difference of arrival (TDoA), i.e., the difference of the reception timestamps has to be corrected for the different transmission times.

Therefore, all the messages contain their time of transmission ($t_{\mathrm{Tx},i}$) expressed in the master time, and its variance. The drift of the anchors local clock with respect to the master time ($\Delta {\dot{t}}_{i}$) is included in the message as well, along with the respective variance. With ideal, non-drifting clocks, the correction of the measured TDoA would be trivial; the difference $d_{i,j}^{\mathrm{ideal}}$ of distances between tag and anchors indexed by *i* and *j* would be

(21) $$d_{i,j}^{\mathrm{ideal}}=c_{0}\;\left(\left(t_{\mathrm{Rx},i}-t_{\mathrm{Tx},i}\right)-\left(t_{\mathrm{Rx},j}-t_{\mathrm{Tx},j}\right)\right),$$

where $t_{\mathrm{Rx}}$ and $t_{\mathrm{Tx}}$ are the reception and transmission timestamps, respectively. The second subscript indicates the anchor of message origin.

**Figure 3:**
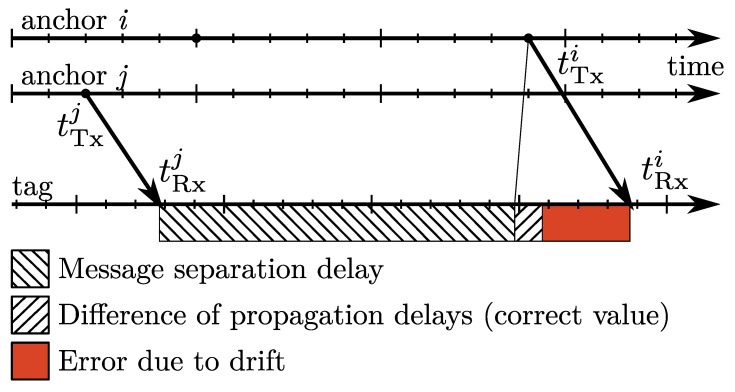
A2T TDoA measurement error due to drift.

Due to an inherent drift between master time and tag time, the transmission and reception time differences are measured using clocks operating at a different pace. The problem is illustrated by an example in Figure 3, the faster pace of the tag clock is visualized by the shorter tick-length for the tag timescale. A number of ticks between the message transmission times $t_{\mathrm{Tx},j}$ and $t_{\mathrm{Tx},i}$ in the master timescale will result in shorter interval in the tag timescale. The difference in propagation delays (and thus distances to anchors) is then corrupted by the error (red rectangle in Figure 3), which is proportional to the relative drift between master and tag clock and to the length of the message separation interval $T_{i,j}$ between transmissions from the anchors indexed by *i* and *j*. The estimate of the drift can be obtained from the CFO measurements $\nu^{\left[MT\right]}$ with respect to the master anchor; the difference of the propagation delays corrected for the drift-related error yields

(22) $$d_{i,j}=c_{0}\;\left(\left(t_{\mathrm{Rx},i}-t_{\mathrm{Tx},i}\right)-\left(t_{\mathrm{Rx},j}-t_{\mathrm{Tx},j}\right)-{\bar{\nu }}^{\left[MT\right]}\;T_{i,j}\right).$$

In order to reduce the effect of the CFO measurement noise on the positioning, the average value of CFO among all anchors received in the particular epoch ${\bar{\nu }}^{\left[MT\right]}$ is utilized. Since the anchor local clocks are not physically synchronous with the master clock, result (20) is employed to obtain the CFO with respect to master anchor. The average CFO is

(23) $${\bar{\nu }}^{\left[MT\right]}=\frac{1}{M}\sum_{l}^{M}\nu_{l}^{\left[MT\right]}\approx \frac{1}{M}\sum_{l}^{M}\left(\nu_{l}^{\left[ST\right]}+\Delta {\dot{t}}_{l}^{\left[\mathrm{MS}\right]}\right),$$

where the *M* available anchors are indexed by *l*. Symbol $\Delta {\dot{t}}_{l}^{\left[\mathrm{MS}\right]}$ denotes estimated bias drift between slave anchor *l* and master, $\nu_{l}^{\left[ST\right]}$ is the tag CFO estimate with respect to slave anchor and $\nu_{l}^{\left[MT\right]}$ is the CFO with respect to master anchor.

It should be pointed out that constant drift is assumed for the interval $T_{i,j}$; typically, the separation interval is several milliseconds and thus, the assumption is valid. In [25], it has been shown that either difference in transmission times or difference in reception times can be used for separation interval computation:

(24) $$T_{i,j}=t_{\mathrm{Tx},i}-t_{\mathrm{Tx},j}=t_{\mathrm{Rx},i}-t_{\mathrm{Rx},j}.$$

With the corrected measurements $d_{i,j}$, the tag’s position in Cartesian coordinates $r\;=\;{\left[\begin{array}{ccc} x & y & z \end{array}\right]}^{T}$ can be estimated by the means of nonlinear least-squares (NLSQ) solver. The problem can be formulated as

(25) $$\min_{r}f\left(r\right)=\min_{r}\sum^{M}{∥d_{i,j}-g_{i,j}\left(r\right)∥}^{2},$$

where $d_{i,j}$ are drift-corrected TDoA measurements according to (22), $g_{i,j}$ is the nonlinear measurement model that estimates TDoA distance from anchors *i* and *j*, located at positions $r_{i}$ and $r_{j}$, respectively, to the tag at position r

(26) $$g_{i,j}\left(r\right)=∥r-r_{i}∥-∥r-r_{j}∥.$$

A point r, where the function (25) reaches a (local) minimum, can be found iteratively with the weighted Levenberg–Marquardt method [27,28,29]. The L-M method is known for its fast convergence [27,28] as it is able to behave either more as a Gauss–Newton method (faster, higher risk of divergence) or a gradient descent method (slower, lower risk of divergence). Such properties are achieved by coupling the two behaviors by carefully manipulating the value of the damping coefficient $\lambda_{k}$.

The L-M method computes the new estimate r[k+1] from the previous one r[k], starting from the initial guess r[0]. The estimate update equation is [29,30]

(27) $$r\left[k+1\right]=r\left[k\right]-{\left(G_{k}^{T}WG_{k}+\lambda_{k}\;\cdot \;\mathrm{Diag}\left(G_{k}^{T}WG_{k}\right)\right)}^{-1}G_{k}^{T}W\left(d-g\left[k\right]\right),$$

where vectors d and g(r[k]) are collated TDoA distance measurements and the respective modeled measurements, matrix W is the measurement weighting matrix, operator Diag(·) clears the off-diagonal elements of the input matrix and $G_{k}$ is matrix of first derivatives of vector g(r[k]) evaluated at point r[k]

(28) $$G_{k}={\left.\frac{\partial g(r)}{\partial r}\right|}_{r[k]}.$$

The matrix $G_{k}$ is a 3×M matrix, where each row is equal to

(29) $$\frac{\partial g_{i,j}\left(r\right)}{\partial r}={\left(\frac{r-r_{i}}{∥r-r_{i}∥}-\frac{r-r_{j}}{∥r-r_{j}∥}\right)}^{T}={\left(1_{i}-1_{j}\right)}^{T},$$

where $1_{i}$ and $1_{j}$ are unit vectors pointing in the direction of user’s estimated position from the anchor’s *i* and *j* position, respectively.

The weight matrix is the inverse of the TDoA measurement covariance matrix $\Phi_{d}$ [29], while the TDoA covariance matrix is obtained by transformation of the time of arrival measurement (ToA) covariance matrix $\Phi_{t}$ with combination matrix D

(30) $$W=\Phi_{d}^{-1}={\left(D\;\cdot \;\Phi_{t}\;\cdot \;D^{T}\right)}^{-1}.$$

The ToA measurement covariance matrix $\Phi_{t}$ is a diagonal matrix with the diagonal elements $\mathrm{var}(t_{i})$ equal to the sum of the variances of ToA measurement $t_{\mathrm{Rx},i}$, transmission time $t_{\mathrm{Tx},i}$ and CFO measurement $\nu_{i}$

(31) $$\mathrm{var}\left(t_{i}\right)=\mathrm{var}\left(t_{\mathrm{Tx},i}\right)+\mathrm{var}\left(t_{\mathrm{Rx},i}\right)+\mathrm{var}\left(\nu_{i}\right).$$

The ToA $\mathrm{var}(t_{\mathrm{Rx},i})=150$ ps (see Section 2) and CFO variances $\mathrm{var}(\nu_{i})$ are constant, while the actual value of $\mathrm{var}(\nu_{i})$ depends on the method of CFO measurement (see Section 3.3). The transmission time variance $\mathrm{var}(t_{\mathrm{Tx},i})$ is estimated and included in the message transmitted by the anchor *i*.

Finally, the combination matrix D is a (M−1)×M matrix that combines the ToA measurements into TDoA measurements. Each row of the matrix contains exactly one +1 and one −1 values, the rest is zero. By multiplying a vector of *M* ToA measurements by matrix D a vector of M−1 TDoA measurements is obtained. The exact format of the matrix is not given, however its choice does affect the hyperboloids used for the estimation and thus the achievable estimation accuracy (Dilution of Precision). The following is an example of a combination matrix

(32) $$D=\left[\begin{array}{cccc} 1 & -1 & \; & 0\\ \; & \ddots & \ddots \\ 0 & \; & 1 & -1 \end{array}\right].$$

The covariance of the final estimate $\Phi_{r}$, given by the described process, can be computed from the jacobian $G_{K}$ from the last algorithm iteration *K* [29]

(33) $$\Phi_{r}={\left(G_{K}^{T}WG_{K}\right)}^{-1}.$$

## 5. Experimental Results

The experimental evaluation of the CFO-aided A2T TDoA positioning consists of measurements in 16 locations within the laboratory’s UWB testbed. We acknowledge that the testbed setup and statistical evaluation is almost identical to the one used in [25], in order to maintain comparability with the results with other positioning methods.

### 5.1. Testbed Description and Data Acquisition

**Figure 4:**
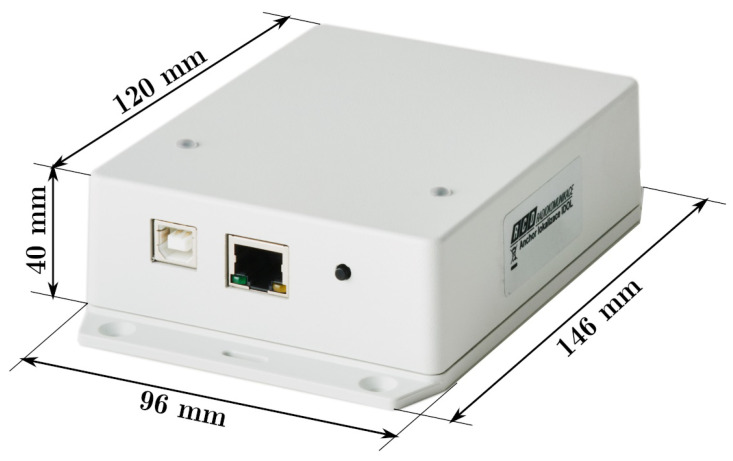
Detailed photo of anchor with dimensions.

**Figure 5:**
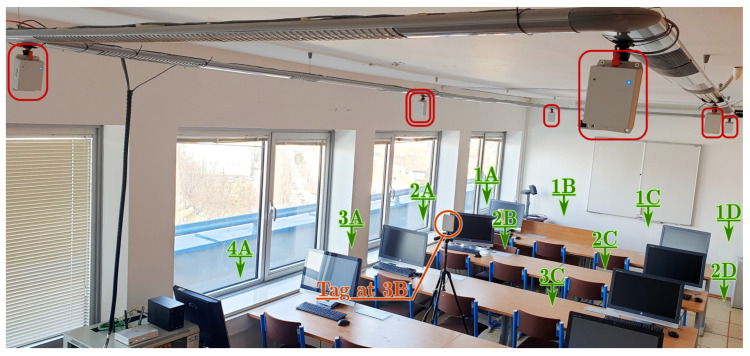
Testbed photography; anchors are highlighted by red rectangles (master anchor by double rectangle); visible test locations marked by green arrows; tag is highlighted by orange circle.

The testbed is a room of dimensions 4.4 by 9.7 m with a ceiling height 2.75 m. Six UWB anchors were mounted on the bottom of a suspended ceiling light, their antennas were located between 2.52 m to 2.57 m above the floor. The anchors were powered and connected to the controlling computer by means of Ethernet cabling with Power-over-Ethernet capability. The detail of the anchor device with its key dimensions is provided in Figure 4. It is worth noting that identical hardware was used as the tag. An annotated photo of the testbed during measurement is provided in Figure 5.

**Figure 6:**
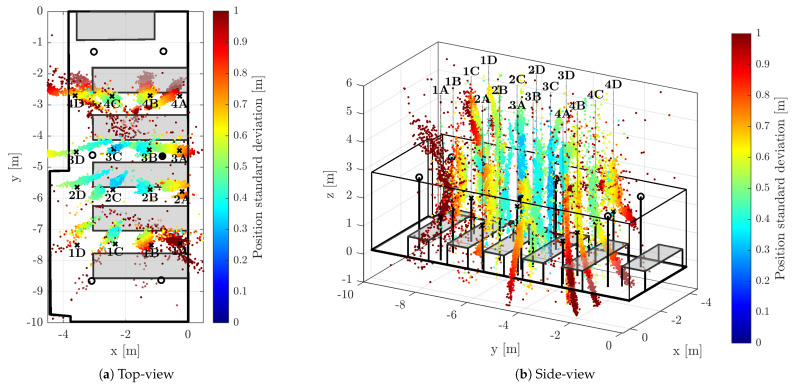
3D Position estimates obtained with TDoA correction based on RTTO CFO ($\nu_{\mathrm{RTTO}}$).

**Figure 7:**
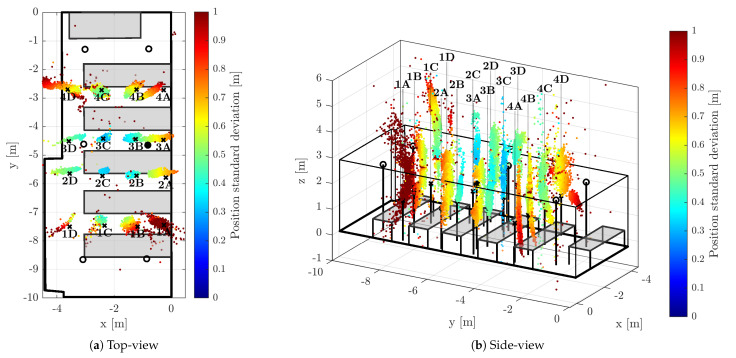
3D Position estimates obtained with TDoA correction based on $C_{int}$ CFO ($\nu_{\mathrm{CINT}}$).

**Figure 8:**
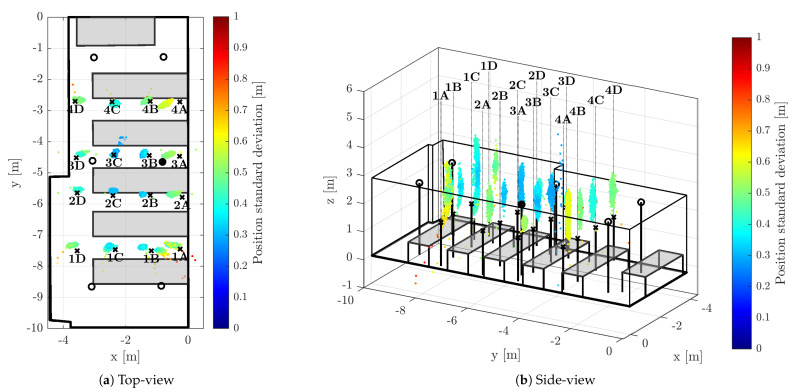
3D Position estimates obtained using EKF with clock drift tracking.

The positions of the anchors were precisely surveyed by a point-to-point laser distance measurement device. The anchors were synchronized by means of algorithm described within Section 2; the master anchor is depicted by a filled circle in the Figure 6, Figure 7 and Figure 8, hollow circle is used for the slave anchors.

During the test runs, all messages received by the tag were stored, including the necessary timestamps and CFO estimates obtained by both methods (RTTO and $C_{int}$). Consequently, all the methods of interest are evaluated at once, inherently under identical conditions and using the same message data and timestamps. The 16 measurement points were organized in a 4-by-4 grid; the points are identified by a number indicating row and letter indicating column, when observed from above. The point 1A is located in the bottom-right corner of Figure 6a, Figure 7a and Figure 8a, the point 4D is in the top-left corner. The tag was mounted on a tripod stand approximately 1.55 m above the floor; the ground-truth position was surveyed by a point-to-point laser distance measurement device at each location.

At each point, the positioning and CFO data were collected for at least 5 min. The synchronization/positioning messages were transmitted with period 100 ms, and thus data from over 3000 epochs were recorded at each point; in total, measurements from 49,666 epochs were collected.

The acquired measurements (timestamps and CFO measurements) were processed independently by the three anchor-to-tag (A2T) TDoA positioning algorithms that were evaluated:

1. CFO-aided A2T TDoA, Levenberg–Marquardt epoch-by-epoch solution; CFO is based on RTTO measurement—$\nu_{\mathrm{RTTO}}$, see (17)
2. CFO-aided A2T TDoA, Levenberg–Marquardt epoch-by-epoch solution; CFO is based on carrier-integrator measurement—$\nu_{\mathrm{CINT}}$, see (18)
3. EKF-based A2T TDoA; drift is estimated and corrected—method described in [25]

The positioning has been performed both in 3D (spatial) and 2D (planar); the results are provided in Section 5.3 and Section 5.4, respectively.

### 5.2. Statistical Evaluation

Before evaluating the accuracy, the position estimates are validated. Positioning results that failed to converge to a sufficiently precise solution are discarded in order to avoid distortion of the results by obviously unreliable values. Such outlier is detected when the absolute value of any position coordinate exceeds 100 m; similarly, an unreliable value is detected when any position coordinate variance exceeds $1\times 10^{4}$ m${}^{2}$ or if “not a number” value occurs either within the position vector or its covariance matrix. The percentage of valid estimates for each of the evaluated algorithms will be stated in their related sections. In the further description of the statistical evaluation, we will denote the number of valid epochs as $N_{v}$.

The non-weighted and weighted means of position estimates ($\bar{r}$ and ${\bar{r}}_{w}$, respectively) for each measurement location are computed as [31]

(34) $$\begin{array}{cc} \bar{r} & =\frac{1}{N_{v}}\sum_{i=0}^{N_{v}-1}r_{i}, \end{array}$$

(35) $$\begin{array}{cc} {\bar{r}}_{w} & =\frac{1}{\sum_{i=0}^{N_{v}-1}w_{i}}\sum_{i=0}^{N_{v}-1}w_{i}r_{i}. \end{array}$$

The weight $w_{i}$ for each epoch is computed as

(36) $$w_{i}=\frac{1}{\mathrm{tr}\left(\Phi_{r,i}\right)}=\frac{1}{\sigma_{rx,i}^{2}+\sigma_{ry,i}^{2}+\sigma_{rz,i}^{2}},$$

where $\Phi_{r,i}$ is the covariance matrix of estimated position in epoch *i*.

The mean values are used when evaluating the mean error of the position estimation:

(37) $$\begin{array}{cc} {\bar{\epsilon }}_{3D} & =∥\bar{r}-r_{ref}∥ \end{array}$$

(38) $$\begin{array}{cc} {\bar{\epsilon }}_{w3D} & =∥{\bar{r}}_{w}-r_{ref}∥. \end{array}$$

Symbol $r_{ref}$ denotes the ground truth position vector. Note that only the first two elements of the vectors are used when evaluating the 2D (horizontal) error.

The spread of the estimates is quantified by means of the unweighted and weighted covariance matrices, Φ and $\Phi_{w}$:

(39) $$\begin{array}{cc} \Phi & =\frac{1}{N_{v}-1}\sum_{i}\left(r_{i}-\bar{r}\right){\left(r_{i}-\bar{r}\right)}^{T}, \end{array}$$

(40) $$\begin{array}{cc} \Phi_{w} & =\frac{\sum_{i}w_{i}}{{\left(\sum_{i}w_{i}\right)}^{2}-\sum_{i}w_{i}^{2}}\sum_{i}w_{i}\left(r_{i}-{\bar{r}}_{w}\right){\left(r_{i}-{\bar{r}}_{w}\right)}^{T}. \end{array}$$

The standard deviations in 3D ($\sigma_{3D}$) and 2D ($\sigma_{2D}$) are then obtained from the covariance matrices as

(41) $$\begin{array}{cc} \sigma_{3D} & =\sqrt{\mathrm{tr}(\Phi )} \end{array}$$

(42) $$\begin{array}{cc} \sigma_{2D} & =\sqrt{\Phi_{1,1}+\Phi_{2,2}}, \end{array}$$

and identically for the weighted values.

The 3D RMS (root mean square) and WRMS (weighted root mean square) are evaluated as

(43) $$\begin{array}{cc} {\mathrm{RMS}}_{3D} & =\sqrt{\frac{1}{N_{v}}\sum_{k=0}^{N_{v}-1}{∥r_{k}-r_{ref}∥}^{2}}, \end{array}$$

(44) $$\begin{array}{cc} {\mathrm{WRMS}}_{3D} & =\sqrt{\frac{1}{\sum_{k=0}^{N_{v}-1}w_{k}}\sum_{k=0}^{N_{v}-1}w_{k}{∥r_{k}-r_{ref}∥}^{2}}. \end{array}$$

Note that 2D (horizontal) RMS and WRMS, are obtained with similar formulas, where only the first two elements of the position vector are taken into account. Inherently, the corresponding covariance matrix is truncated to size 2×2.

All the metrics are evaluated separately for each of the 16 measurement points 1A to 4D. In order to obtain the results characterizing the whole test (in the tables marked as TOT.), the quadratic mean is employed:

(45) $$\begin{array}{cc} \sigma_{3D,\mathrm{TOT}} & =\sqrt{\frac{1}{K}\sum_{k=0}^{K-1}\sigma_{3D}^{2}}, \end{array}$$

(46) $$\begin{array}{cc} {\bar{\epsilon }}_{3D,\mathrm{TOT}} & =\sqrt{\frac{1}{K}\sum_{k=0}^{K-1}\epsilon_{3D,k}^{2}}, \end{array}$$

(47) $$\begin{array}{cc} {\mathrm{RMS}}_{3D,\mathrm{TOT}} & =\sqrt{\frac{1}{K}\sum_{k=0}^{K-1}{\mathrm{RMS}}_{3D,k}^{2}}. \end{array}$$

The individual measurements from the set of size K=16 are indexed by *k*. The 2D metrics and the weighted metrics are totaled in an identical way. This approach is insensitive to the number of valid measurements on each of the measurement locations.

### 5.3. 3D Positioning Results

In the spatial positioning (3D) the height of the tag was estimated along with the horizontal coordinates. The accuracy in the vertical direction was expected to be poor, since the constellation of the anchors is rather flat, and therefore high VDOP (vertical dilution of precision) deteriorates the accuracy. Although inaccurate, the vertical coordinate may be treated as nuisance parameter, while the more accurate horizontal coordinates are still useful.

The results of the three A2T TDoA positioning methods were compared both qualitatively and quantitatively.

Figure 6, Figure 7 and Figure 8 provide visual representations of the results for all 16 measurement points. The (a) subfigures show the testbed and constellation from above, the (b) subfigures present the identical results viewed from side. The ground-truth positions are marked by the black crosses. In order to improve readability, there are black vertical lines from the crosses (tag ground-truth) and circles (anchors) to the floor level. The position estimates are depicted with colored dots; the color indicates the estimate horizontal standard deviation, i.e., the result of (42) for the particular measurement epoch.

#### 5.3.1. 3D Localization Aided by RTTO-Based CFO

The CFO-aided TDoA with the CFO based on RTTO measurement ($\nu_{\mathrm{RTTO}}$) is rather inaccurate, as can be observed in Figure 6. In the horizontal plane (Figure 6a) the position estimates appear to be spread outwards from the constellation. The side-view (Figure 6b) reveals that the most prominent error is in the vertical domain. The position estimates at each measurement point follow a hyperbolic curve, i.e., the line of constant TDoA. The vertical character of the position uncertainty is caused by the flatness of the anchor constellation and thus, high vertical dilution of precision (VDOP). The inaccuracy and spread is caused mostly by the poor accuracy of the RTTO-based CFO.

The inaccurate RTTO-based drift correction also reduces the convergence rate of the L-M algorithm, in fact, only 73.1% of the solutions passed the validation criteria described in Section 5.2. The presence of a number of highly inaccurate position estimates is apparent from the non-weighted statistics that are available in the left part of Table 1. It is apparent that the most of the horizontal RMS error (${\mathrm{RMS}}_{2}D$) is due to the spread of the estimate quantified by the standard deviation $\sigma_{2}D$; the mean of the error (${\bar{\epsilon }}_{2}D$) is approximately order-of-magnitude smaller than the standard deviation. The spatial RMS error (${\mathrm{RMS}}_{3}D$) is typically double the horizontal RMS, confirming that the error in the vertical dimension is the largest one.

Since the highly inaccurate position estimates usually feature exceptionally large estimated standard deviations, the corresponding weighted statistics (left part of Table 2) are substantially more favorable than the unweighted ones. In particular, the horizontal ${\mathrm{WRMS}}_{2}D$ is 56.6 cm, i.e., almost 10-times lower than the unweighted RMS. The weighted standard deviation is also comparable to the weighted mean error. However, from comparison of ${\mathrm{WRMS}}_{3}D$ and ${\mathrm{WRMS}}_{2}D$ it is obvious that the significant vertical error is eminent for all position estimates; the difference is even more distinct than for the non-weighted RMS metrics.

#### 5.3.2. 3D Localization Aided by Carrier-Integrator-Based CFO

The CFO-aided TDoA with the CFO based on carrier-integrator measurement ($\nu_{\mathrm{CINT}}$) provides better results, indeed. Although the outward spread of the position estimates in Figure 7a is similar to the previous result in shape and direction, the magnitude of the error is substantially lower. From Figure 7b it is obvious that even the vertical error is lower in comparison with the RTTO CFO-aided result; the estimates are more concentrated around the hyperbolic segments, the vertical spread is lower as well.

Undoubtedly, the quantitative results confirm better performance when utilizing $C_{int}$-based CFO. In particular, 80.1% of the position estimates passed the validation criteria from Section 5.2. Still, the most of the error is caused by the spread of the measurements, see the unweighted statistics in the central part of Table 1. The improvement with respect to the RTTO-based CFO varies depending on the test point; however, overall standard deviation and RMS errors for the $C_{int}$-based estimates are generally lower by approximately one third. The total mean of the error (${\bar{\epsilon }}_{2}D$) is rather similar for both CFO-aided approaches; however, the values vary significantly for each test point, there are several test points where the mean error is higher for the $C_{int}$-based estimates.

The improvement is similar for the weighted metrics, as reported in the central part of Table 1. As the influence of highly inaccurate measurements is suppressed, the horizontal weighted standard deviation and WRMS is a few decimeters for all measurement points when $C_{int}$-based CFO is utilized. The effect of the choice of CFO source does not possess a significant effect on the weighted mean horizontal error ${\bar{\epsilon }}_{w2D}$. When $C_{int}$-based CFO is used, the weighted mean horizontal error is similar in magnitude to the weighted standard deviation $\sigma_{w2D}$.

#### 5.3.3. EKF-Based 3D Localization

The extended Kalman-filter-based solution is used as a benchmark; the achieved results are consistent with the previous evaluation in [25]. The horizontal spread of the estimates is very low, mostly smaller than the offset from the ground-truth position, see Figure 8a. The larger spread in the vertical dimension is documented in Figure 8b; additionally, there is a significant bias towards the anchor constellation plane in the vertical dimension.

There is only a negligible difference between the unweighted and weighted quantitative results for the EKF-based solution that are provided in the right part of Table 1 and Table 2. Unlike for the CFO-aided solutions, the bias of the estimate, i.e., mean error ${\bar{\epsilon }}_{2}D$ is the more prominent contributing factor to the RMS error than the spread quantified by the standard deviation $\sigma_{3D}$. Indeed, the horizontal standard deviation is in the centimeter level for all test points except location 1A. The high value of spatial ${\mathrm{RMS}}_{3}D$ is mostly caused by the apparent vertical bias, since the spatial standard deviation $\sigma_{3D}$ (not included in the tables) of the EKF-based solution is in the order of lower decimeters, typically.

It is necessary to note that 99.8% of position estimates passed the validation criteria; however, comparison of the validation pass-rate of the between EKF-based and epoch-by-epoch solutions is rather unjust due to their different nature.

### 5.4. 2D Positioning Results

**Figure 9:**
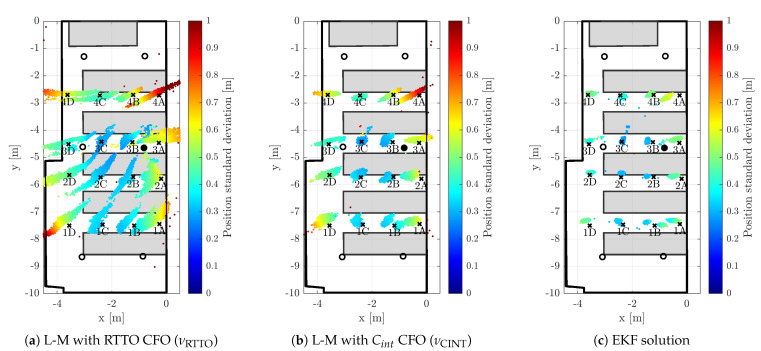
2D Position estimates obtained using EKF with clock drift tracking.

In this case a purely planar (2D) solution is obtained, i.e., vertical coordinates of both the tag and the anchors are neglected. The unequal heights of tag and the anchors are not compensated in any way, since the height of the tag is considered unknown. Consequently, the neglection of the vertical coordinates contributes to the overall positioning error of the planar (2D) solution. On the contrary, there is no error contribution related to high VDOP and thus the 2D solution may provide more accurate results than the 3D solution in certain cases. The visualization of the results of the three evaluated algorithms in 2D are provided side by side in Figure 9.

#### 5.4.1. 2D Localization Aided by RTTO-Based CFO

Although the 2D TDoA with the CFO aiding based on RTTO measurement ($\nu_{\mathrm{RTTO}}$) is more accurate than its 3D counterpart, the accuracy is still rather poor. The planar positioning results can be seen in Figure 9a. It is necessary to note that neglecting the vertical coordinate leads to substantially higher validation pass-rate, which reaches 98.5%. The non-weighted statistics of the 2D solution with RTTO-based CFO aiding are provided in the left part of Table 3. It is apparent that in total the horizontal standard deviation ($\sigma_{2}D$) and RMS error (${\mathrm{RMS}}_{2}D$) are approximately a third of the values obtained by the 3D solution (see Table 1).

The improvement of 2D solution is less prominent in case of the weighted horizontal standard deviation ($\sigma_{w2D}$) and RMS error (${\mathrm{WRMS}}_{2}D$), see Table 4. In comparison with the 3D solution we observe approximately 42% improvement of $\sigma_{w2D}$ and 42% in ${\mathrm{WRMS}}_{2}D$. Such results suggest that the 2D solution leads to more stable convergence and reduces the number of outliers.

#### 5.4.2. 2D Localization Aided by Carrier-Integrator-Based CFO

The reduction of the solution to two dimensions is also beneficial when CFO aiding is based on carrier integrator measurement ($\nu_{\mathrm{CINT}}$). Indeed, the validation criteria, as described in Section 5.2, were passed in 99.5% of epochs. The results are visualized in Figure 9b; apparently, the carrier integrator CFO-aided positioning is substantially more accurate than the solution with the RTTO-based CFO aiding.

The accuracy is quantified by the non-weighted statistics provided in central part of Table 3. The unweighted statistics suggest major improvement in terms of accuracy; horizontal standard deviation ($\sigma_{2}D$) is 84% lower and RMS error (${\mathrm{RMS}}_{2}D$) is 82% lower with respect to the 3D solution that estimates the vertical coordinate as well. The improvement with respect to the RTTO-based 2D solution is 65% in terms of $\sigma_{2}D$ and 61% when comparing to ${\mathrm{RMS}}_{2}D$.

The weighted statistics in the central part of Table 4 confirm the obvious improvement, which nonetheless, is not as impressive as in the non-weighted case. The weighted standard deviation ($\sigma_{w2D}$) is 51% lower and ${\mathrm{WRMS}}_{2}D$ is 10% lower than those achieved with the 3D solution. Clearly, the 3D solution contained substantial amount of estimates with high error and very low weight (low confidence). In comparison with the RTTO-based 2D solution there is 60% and 35% improvement in ($\sigma_{w2D}$) and ${\mathrm{WRMS}}_{2}D$, respectively.

It is worth noting that the difference in mean error of the position estimation regardless weighting (i.e., ${\bar{\epsilon }}_{2}D$ and ${\bar{\epsilon }}_{w2D}$) is far less prominent than the change in standard deviation or RMS error.

#### 5.4.3. EKF-Based 2D Localization

The EKF-based 2D solution completes the graphical results in Figure 9c. There is no substantial performance difference between the 2D and 3D solution when the EKF is used to mitigate the clock drift effect on the A2T TDoA positioning. It is acknowledged that the validation pass-rate is identical to the 3D solution, i.e., 99.8%. The weighted and non-weighted statistical results are available in the right part of Table 3 and Table 4. The horizontal-plane statistics (i.e., $\sigma_{2}D$, ${\bar{\epsilon }}_{2}D$, ${\mathrm{RMS}}_{2}D$) and their weighted counterparts differ mostly by several millimeters regardless of whether a 2D or 3D solution is employed.

## 6. Discussion

The presented results clearly show that it is possible to utilize CFO measurements to correct anchor-to-tag TDoA measurements, and obtain position on an epoch-by-epoch basis. However, the CFO measurements (both RTTO-based and carrier-integrator-based) are substantially less accurate than the clock drift estimate obtained by Kalman filtering from the longer observation of timestamps. Naturally, the CFO-aided A2T TDoA suffer from substantially poorer accuracy. Notably, the least accurate RTTO-based CFO bears minimal utility as an aid to positioning. The CFO obtained from the carrier integrator is more accurate, and thus the positioning results are more favorable. It is worth mentioning that the more accurate $C_{int}$-based CFO yields a higher percentage of valid solutions than the RTTO-based CFO. Therefore, it is recommended to utilize the $C_{int}$-based CFO only, even though it is slightly more difficult to obtain from the transceiver.

Due to the geometry of the anchor constellation, the positioning performance is inherently better for the test points located in the central part of the constellation rather than on the edges, where the poor geometry emphasizes the inaccuracies in the TDoA measurements; this is certainly valid even for the EKF-based solution.

The epoch-by-epoch CFO-aided estimates are not affected by any underlying motion and drift model, unlike the EKF solution [25]. Inherently, the non-filtered position estimates seem to be more spread, since there is no assumption of inter-epoch dependency. Nonetheless, it is apparent that the major contributing factor to the inaccuracy of the CFO-aided solutions is the poor accuracy of the CFO estimate in comparison to the EKF-based drift estimate.

Graphical results and both the weighted and non-weighted statistical metrics suggest that the horizontal position estimate biases (quantified by ${\bar{\epsilon }}_{w2D}$ or ${\bar{\epsilon }}_{2D}$) are quite similar for all presented solutions. The bias errors that are common to all solutions are probably caused by inaccuracies in anchor constellation survey and internal delays calibration. Due to the higher spread (quantified by $\sigma_{w2D}$ or $\sigma_{2D}$) of the CFO-aided solutions, the impact of such systematic errors on the overall horizontal RMS accuracy is not consequential. Nonetheless, they represent a major contribution to the horizontal RMS of the EKF-based solution.

All the presented 3D positioning results suffer from higher spread and substantial bias in the vertical direction. Such bias is more difficult to observe for the CFO-aided solutions due to higher standard deviations. Nonetheless, such behavior is caused mostly by the flat constellation of anchors, and thus, the vertical dilution of precision (VDOP) is rather high. The position estimates are biased towards the plane formed by the anchors (see Figure 8b). The performance in the vertical dimension could be improved by placing several anchors closer to the floor level; however, such placement is rather impractical for most scenarios due to obstructions in the signal path.

It is also possible to neglect the vertical coordinate completely and solve the whole problem in plane, i.e., in two dimensions only (2D). The presented results indicate substantial performance improvement of both 2D CFO-aided solutions with respect to the 3D solutions obtained from the identical TDoA measurements. Not only is the accuracy better, but especially the solution validation pass-rate is higher for the 2D solution; it reaches 98.5% and 99.5% for the CFO based on RTTO and carrier integrator, respectively.

Alternatively, if the height of the tag is known, a soft constraint may be introduced into either the least-squares or EKF solution, see e.g., [32]. An evaluation on the impact of the constraints or solution dimension reduction on the horizontal and overall accuracy of the CFO-aided solution is beyond the scope of this article. The effect of soft constraints on EKF solution has been already described in [25].

Although the CFO-aided A2T TDoA solutions are less accurate in comparison with the EKF-based solution, they are useful due to their unique features. Since the localization is performed on an epoch-by-epoch basis, it can be utilized by low-power devices that do not localize themselves continuously, but rather infrequently (e.g., once per minute or less). For such devices, it is not feasible to observe numerous epochs in order to obtain accurate results from the EKF. With the CFO-aided solution, it is necessary to turn the receiver on for a single epoch only, and therefore optimize the energy consumption of the device. Alternatively, the CFO-aided position estimate can be utilized to trigger accurate EKF-based positioning only in certain areas and conserve resources when accurate positioning is not necessary. It is worth mentioning that both the CFO-aided L-M solutions and the EKF solution can be implemented in low-cost devices.

Utilizing either of the CFO estimates directly in the EKF as a drift measurement is not beneficial due to its poor accuracy. Nevertheless, CFO and CFO-based position estimates can be used as the initial condition to the EKF. The CFO-based drift value may be also utilized to detect a failure or divergence of the EKF in devices that estimate the position frequently and continuously, and improve the robustness of the positioning system.

## 7. Conclusions

The presented results confirm that it is possible to use carrier-frequency offset (CFO) to compensate for tag clock drift when estimating tag’s position. Since positioning messages are sent by the synchronized network of anchors (A2T TDoA), and the tag only receives, the number of possible users is virtually unlimited. The algorithm for correcting the TDoA measurements by means of the CFO observations with respect to multiple transmitting anchors was described and experimentally evaluated. Although the accuracy of the CFO-aided approach does not reach the one of the EKF solution, it is able to operate on epoch-by-epoch basis, and thus does not require continuous operation to converge. Such characteristic may be useful especially for devices optimized for long battery life, which do not require frequent position updates.

In total, two sources of the CFO-aiding were evaluated; the utilization of RTTO-based CFO value yields inaccurate results that bare minimal utility without further filtering. According to the observations, only the use of carrier-integrator-based CFO measurement is feasible for positioning. Furthermore, it was shown that when CFO-aiding is used, it is beneficial to neglect vertical coordinate and solve the positioning problem in plane (2D) rather than in space (3D). With this approach, we were able to achieve horizontal ${\mathrm{WRMS}}_{2}D$ of 30.3 cm when the height coordinate was neglected and ${\mathrm{WRMS}}_{2}D$ of 34.0 cm when estimating the height as well.

## Figures and Tables

**Table 1:** Non-weighted statistics of 3D position estimates (all values in centimeters).

	L-M with RTTO CFO ($\nu_{\mathrm{RTTO}}$)				L-M with $C_{\mathrm{int}}$ CFO ($\nu_{\mathrm{CINT}}$)				EKF Solution			
ID	$\sigma_{2}D$	${\bar{\epsilon }}_{2}D$	WRMS2D	WRMS3D	$\sigma_{2}D$	${\bar{\epsilon }}_{2}D$	WRMS2D	WRMS3D	$\sigma_{2}D$	${\bar{\epsilon }}_{2}D$	WRMS2D	WRMS3D
1A	649.6	115.9	659.7	1391.5	562.5	13.8	562.5	892.3	23.7	35.2	42.5	105.5
1B	604.7	40.1	605.8	1311.8	584.6	23.8	584.8	732.3	7.0	31.2	32.0	100.5
1C	808.1	58.8	810.0	1398.7	508.8	24.5	509.3	961.0	8.1	22.6	24.0	116.1
1D	1226.4	33.3	1225.8	2009.9	549.3	121.3	560.5	1633.8	8.5	23.2	24.7	99.7
2A	540.5	36.4	541.5	899.0	227.4	26.1	228.9	427.4	7.4	30.2	31.1	99.2
2B	247.5	18.4	248.2	376.9	78.8	17.9	80.8	157.9	4.1	22.6	23.0	101.0
2C	77.6	14.8	79.0	340.7	21.3	16.1	26.6	181.2	5.1	15.6	16.4	84.8
2D	415.3	50.9	418.3	717.9	365.5	17.7	365.8	631.4	6.2	11.3	12.9	53.8
3A	301.0	17.6	301.4	410.3	426.3	36.1	427.7	533.8	8.1	34.4	35.4	55.2
3B	186.5	14.2	187.0	378.6	93.6	8.3	93.9	231.8	6.1	19.0	19.9	101.8
3C	138.2	9.9	138.5	383.9	81.2	6.9	81.4	396.0	7.0	4.4	8.3	100.4
3D	191.8	18.5	192.7	438.0	21.3	20.3	29.4	162.3	5.6	17.9	18.7	45.1
4A	218.8	32.8	221.2	402.9	16.9	49.0	51.8	74.7	9.2	46.0	46.9	131.9
4B	188.3	18.3	189.1	435.2	14.0	21.2	25.4	72.4	7.0	12.8	14.6	99.2
4C	233.9	22.5	234.9	808.7	73.0	5.6	73.2	368.9	7.0	8.8	11.3	99.4
4D	403.9	52.2	407.2	1280.8	74.8	12.0	75.7	251.6	7.5	13.8	15.7	99.5
TOT.	497.4	43.1	499.0	953.0	318.2	37.5	320.1	627.3	9.0	24.4	26.0	95.9

**Table 2:** Weighted statistics of 3D position estimates (all values in centimeters).

	L-M with RTTO CFO ($\nu_{\mathrm{RTTO}}$)				L-M with $C_{\mathrm{int}}$ CFO ($\nu_{\mathrm{CINT}}$)				EKF Solution			
ID	$\sigma_{w2D}$	${\bar{\epsilon }}_{w2D}$	${\mathrm{WRMS}}_{2}D$	${\mathrm{WRMS}}_{3}D$	$\sigma_{w2D}$	${\bar{\epsilon }}_{w2D}$	${\mathrm{WRMS}}_{2}D$	${\mathrm{WRMS}}_{3}D$	$\sigma_{w2D}$	${\bar{\epsilon }}_{w2D}$	${\mathrm{WRMS}}_{2}D$	${\mathrm{WRMS}}_{3}D$
1A	127.5	10.5	98.4	344.4	64.6	13.1	52.5	244.2	24.5	35.4	42.8	106.7
1B	83.8	10.5	49.5	293.1	47.0	22.1	37.2	217.7	10.2	29.4	31.6	98.6
1C	82.3	26.8	51.4	317.7	39.2	30.0	37.2	287.2	6.7	22.7	23.4	116.5
1D	90.6	48.7	88.7	306.5	49.1	43.7	58.6	261.5	9.1	23.0	24.0	99.9
2A	42.3	18.8	46.1	204.8	24.2	22.5	35.7	161.1	7.2	28.9	31.1	98.6
2B	25.2	20.5	28.7	209.3	8.6	15.2	18.8	44.2	4.1	22.5	22.9	99.8
2C	36.2	15.9	34.4	194.4	10.6	15.4	18.7	184.5	4.8	15.6	16.3	81.8
2D	36.6	36.7	50.3	117.3	19.4	11.1	22.0	121.5	5.4	11.3	12.4	52.9
3A	32.4	30.6	48.8	98.2	23.0	23.6	35.2	94.0	7.5	34.6	35.4	55.2
3B	20.3	14.8	25.0	79.8	10.3	6.8	15.0	47.3	6.0	18.1	20.0	102.8
3C	34.3	2.6	26.4	173.9	14.4	1.4	10.7	127.2	8.0	4.0	8.1	102.5
3D	52.5	33.8	69.1	162.0	17.4	23.5	29.1	161.2	4.7	17.9	18.5	44.7
4A	46.1	42.8	64.4	170.8	15.8	46.5	53.9	65.8	7.9	45.2	46.9	137.2
4B	34.7	22.6	32.2	149.0	12.1	22.0	25.4	61.8	6.0	12.8	14.0	99.1
4C	68.9	24.2	49.3	180.7	18.2	9.1	16.5	89.1	6.3	8.7	10.6	99.3
4D	105.2	27.8	76.3	212.0	44.8	17.0	27.8	114.3	7.5	13.9	15.0	100.0
TOT.	65.1	27.0	56.6	214.8	31.0	23.4	34.0	161.5	9.1	24.0	25.8	96.3

**Table 3:** Non-weighted statistics of 2D position estimates (all values in centimeters).

	L-M with RTTO CFO ($\nu_{\mathrm{RTTO}}$)			L-M with $C_{\mathrm{int}}$ CFO ($\nu_{\mathrm{CINT}}$)			EKF Solution		
ID	$\sigma_{2}D$	${\bar{\epsilon }}_{2}D$	WRMS2D	$\sigma_{2}D$	${\bar{\epsilon }}_{2}D$	WRMS2D	$\sigma_{2}D$	${\bar{\epsilon }}_{2}D$	WRMS2D
1A	525.5	71.4	530.2	24.2	44.4	50.6	23.8	36.2	43.3
1B	32.8	30.6	44.9	12.8	31.5	34.0	6.2	31.5	32.2
1C	123.8	4.4	123.9	24.4	26.2	35.8	6.6	23.2	24.2
1D	90.9	24.9	94.3	189.8	29.0	192.0	8.4	23.2	24.7
2A	33.8	39.6	52.1	13.4	46.4	48.3	7.2	30.7	31.5
2B	41.3	26.7	49.2	11.7	25.1	27.7	4.1	22.7	23.0
2C	36.8	13.6	39.2	11.2	12.6	16.8	4.9	14.6	15.4
2D	82.9	11.9	83.8	23.5	5.6	24.2	6.2	9.8	11.6
3A	69.4	19.2	72.0	16.6	13.7	21.5	10.1	17.5	20.2
3B	64.9	14.1	66.4	13.6	25.8	29.1	5.3	20.3	21.0
3C	34.6	3.5	34.7	23.4	4.4	23.8	6.6	4.5	8.0
3D	67.5	13.2	68.8	23.1	12.0	26.0	6.4	11.4	13.1
4A	73.4	20.4	76.1	21.4	34.3	40.4	9.2	47.3	48.2
4B	18.8	15.4	24.3	14.9	17.6	23.1	7.0	12.7	14.5
4C	48.7	5.8	49.1	9.7	6.9	11.9	6.9	8.6	11.0
4D	65.9	11.6	66.9	15.2	7.4	16.9	7.2	13.8	15.6
TOTAL	145.6	26.1	147.9	50.6	25.1	56.4	9.0	23.3	25.0

**Table 4:** Weighted statistics of 2D position estimates (all values in centimeters).

	L-M with RTTO CFO ($\nu_{\mathrm{RTTO}}$)			L-M with $C_{\mathrm{int}}$ CFO ($\nu_{\mathrm{CINT}}$)			EKF Solution		
ID	$\sigma_{w2D}$	${\bar{\epsilon }}_{w2D}$	${\mathrm{WRMS}}_{2}D$	$\sigma_{w2D}$	${\bar{\epsilon }}_{w2D}$	${\mathrm{WRMS}}_{2}D$	$\sigma_{w2D}$	${\bar{\epsilon }}_{w2D}$	${\mathrm{WRMS}}_{2}D$
1A	37.3	44.1	57.7	24.9	50.2	56.1	23.4	38.0	44.7
1B	22.3	32.3	39.3	12.7	31.6	34.1	4.2	31.5	31.8
1C	27.1	24.0	36.2	8.1	26.5	27.7	4.7	23.2	23.7
1D	39.7	31.0	50.3	17.1	26.3	31.4	6.7	23.1	24.0
2A	29.0	41.7	50.8	13.2	48.4	50.2	6.7	30.8	31.5
2B	29.3	27.0	39.8	11.7	25.2	27.8	3.7	22.6	22.9
2C	34.1	16.3	37.8	11.1	12.5	16.8	4.7	14.6	15.3
2D	53.7	23.8	58.7	16.2	5.3	17.0	5.5	9.8	11.3
3A	49.9	23.3	55.0	16.2	13.9	21.4	10.3	17.6	20.4
3B	33.3	16.5	37.2	10.7	26.1	28.2	5.2	20.3	21.0
3C	24.0	4.4	24.4	9.0	3.8	9.8	6.5	4.5	7.9
3D	58.3	29.8	65.4	20.9	12.0	24.1	5.6	11.4	12.7
4A	43.3	41.0	59.6	20.8	38.2	43.5	8.8	47.6	48.4
4B	17.7	18.3	25.5	13.1	19.4	23.4	5.9	12.8	14.1
4C	32.6	5.6	33.1	9.6	7.0	11.9	5.8	8.5	10.3
4D	45.3	28.4	53.4	14.8	8.6	17.1	5.9	13.9	15.1
TOTAL	37.8	27.8	46.9	15.1	26.3	30.3	8.4	23.5	25.0

## Data Availability

The recorded data used for the algorithm evaluation are stored in proprietary and confidential format and are therefore not publicly available. However, both the measurement data and positioning results can be provided by the authors upon request.

## Abbreviations

The following abbreviations are used in this manuscript:

A2T	anchor-to-tag
$C_{int}$	Carrier integrator
CFO	Carrier frequency offset
CIR	Channel impulse response
DOP	Dilution of precision
EKF	Extended Kalman filter
GNSS	Global navigation satellite systems
KF	Kalman filter
L-M	Levenberg–Marquardt
NLSQ	Nonlinear least-squares
ppb	Parts per billion
ppm	Parts per million
RMS	Root mean square
RTTO	real-time tracking offset
STD	standard deviation
T2A	tag-to-anchor
TDoA	Time difference of arrival
ToA	Time of arrival
TWR	Two-way ranging
VDOP	Vertical dilution of precision
WRMS	Weighted root mean square
